# Spatio-temporal expansion patterns and influencing factors in China’s new local retail industry: A case study of Luckin Coffee

**DOI:** 10.1371/journal.pone.0320933

**Published:** 2025-07-16

**Authors:** FengXun Qi, WeiShan Qin, Xiaohan Li

**Affiliations:** School of Resource and Environment Engineering, Ludong University, Yantai, China; Alcorn State University, UNITED STATES OF AMERICA

## Abstract

with the increased penetration of the Internet, artificial intelligence, and other technologies, New Retail, an emerging commercial sector in cities, has gained significant momentum. As a new local retail brand in China, Luckin Coffee has actively explored the new retail model and rapidly captured the market. Its expansion strategy and influence mechanisms are worth exploring in depth. Based on point of interest (POI) data of China’s Luckin stores (2018–2023), this study constructs a geographical indicator system and employs spatial analysis and factor detection methods to study the evolution of Luckin Coffee’s resource agglomeration patterns and its formation mechanism. The results indicate the following: (1) Luckin Coffee’s layout in China exhibits a highly concentrated and unbalanced spatial distribution pattern, showing an expansion trend from coastal regions toward inward diffusion, with most stores located in economically developed areas such as urban agglomerations and first- and second-tier cities; (2) Luckin Coffee’s spatial expansion pattern has progressed through the three phases: initially dominated by hierarchical diffusion, then by a combination of hierarchical and contact diffusion, and finally by contact diffusion, (3) the spatial differentiation in the number of Luckin Coffee stores results from the interplay of multiple factors. However, the influence of each factor on spatial differentiation varied significantly. Specifically, urban construction levels and consumption capacity within the second geographic area, along with informatization levels and human capital in the third geographic area, exhibit the greatest explanatory power regarding spatial distribution. (4) The impact of primary influencing factors on Luckin Coffee’s spatial distribution revealed spatial variability, with notable local imbalances. These imbalances are significant, and the analyzed units with relevant influences exhibit block and band aggregation characteristics. This study provides empirical evidence to supplement current research on the spatial expansion patterns and influence mechanisms of New Retail brands in China.

## Introduction

The spatial expansion and competition of enterprises are important topics of interest in geography, economics, and other disciplines. According to Taylor, the process of spatial expansion of enterprises involves crossing the thresholds of three different spatial scales-local, regional, national, and multinational-and undergoing the corresponding organizational transformations [1]. In recent years, new retail models for online and offline integration have developed rapidly. These new retail models entail optimizing and upgrading capital flow, logistics, and information flow within the retail system by leveraging the Internet and advanced technologies, such as big data, artificial intelligence, and Internet of Things [2]. Under the new retail model, business operations have shifted from “bricks + mortar” to “brick, click and mobile” [3]. This transformation in the retail model will have a profound impact on the location choice and diffusion mechanism of retail business. What is the spatial expansion pattern and location selection mechanism of the new retail industry, how does it differ from that of the traditional retail industry, and what new factors affect it?

Throughout the theoretical research on geography, the spatial structure and location choice of retail has been a core topic. Among these, the center-ground theory has an important position in retail space research. According to this theory, retail spaces are mainly concentrated in central cities and metropolitan areas, showing a diffusion pattern from high-grade cities to small- and medium-sized cities [4]. This diffusion reflects the spatial distribution pattern of the headquarters and branches of retail firms, which is usually closely related to the city’s hierarchical order [5]. However, with the continuous development of the retail industry, studies have shown that the distribution pattern of retail spaces has become more complex [6], especially under the interaction of multiple economic and policy factors, and the traditional hierarchical diffusion model is no longer sufficient to explain the current location choice and diffusion mechanism [7–9]. First, the implementation of the open-door policy [10] created new opportunities for retail development in many regions, enabling retail enterprises to gain market share in areas where they did not have an advantage. In addition, geographic rootedness [11] and industry cluster effects [12] play key roles in the retail spatial layout. Studies show that retail firms tend to choose areas with solid industrial foundations and obvious cluster effects to achieve rapid expansion by leveraging existing resources and market networks [13,14]. However, when new industries or business forms emerge, windows of opportunity emerge to break the established path of regional development and enable latecomer regions to access new development opportunities. Technological advances [15], policy support [16], and the strategic behavior of firms [17] may create windows of opportunity, which may in turn affect the location choices of retail firms.

In addition to traditional retailing, e-tailing is a retail format that is more aligned with new retailing. E-tailing involves a blend of online and offline retailing, incorporating both physical and online sales. In contrast, new retailing builds upon e-tailing by integrating “big data” [2]. Therefore, research on e-tailing provides new analytical perspective for understanding the spatial processes of new retail. From a geographic perspective, scholars have increasingly focused on e-tailing in terms of location choice, spatial patterns, influencing factors, and spatial effects [18]. Investigating the spatial differentiation of e-tailing development and its influencing mechanisms across various scales is another crucial area of research. Domestic scholars have analyzed the development of e-tailing at different scales, such as provincial [19], prefectural [20] and county [21], revealing its spatial distribution characteristics and regional differences. The formation and development of e-tailing is driven by several factors, including the level of regional economy [22], Internet infrastructure [23], and policy support [24]. Research on location selection for online stores in four cities-Shanghai, Shenzhen, Tianjin, and Beijing-has indicated that online stores in these cities are primarily concentrated in areas with convenient transportation and developed economy. This concentration suggests that geographic location plays a key role in the spatial distribution of e-commerce [25]. In terms of the spatial diffusion of e-tailing, Anderson proposed the innovation diffusion hypothesis and efficiency hypothesis, arguing that e-tailing development can be regarded as the process of new technologies diffusion within a region [26]. Some studies have also highlighted that the diffusion of e-tailing in China exhibits a “reverse hierarchy” distribution pattern, where emerging retailers prioritize economically backward regions with specific market advantages, a phenomenon challenging traditional location choice theories [27]. Furthermore, the development of e-tailing has also significantly impacted the organization of urban retail space. For instance, it has altered the organization of urban retail spaces, prompting the emergence of new business models and spatial forms [28]. Breaking the spatial limitations of traditional retailing, e-tailing has made retail spaces more flexible and diversified [29], thus contributing to the remodeling of the urban commercial space structure.

Overall, studying location choice and spatial diffusion mechanisms in the retail sector is significant for understanding commercial geographic distribution and urban spatial structures. Despite the valuable findings of existing research, the ongoing advancement of Internet technology has transformed business models from traditional retail to e-commerce and now to a new retail model, resulting in more complex and diverse patterns and mechanisms of retail space. However, by combing through the current research status of the new retail model, it is found that domestic and foreign scholars tend to focus more on studying the connotation and development trends of the new retail model. From the perspectives of customer needs [30], channel integration [31,32], and service experience [33], qualitative analysis methods are used to explore the development prospects of the new retail model. Nevertheless, there is a lack of detailed research on the spatial expansion characteristics, location preferences, layout patterns, and site - selection influencing mechanisms of new retail. Moreover, the corresponding quantitative research methods are absent for support. Therefore, further in - depth research by scholars is still required.

**Fig 1:**
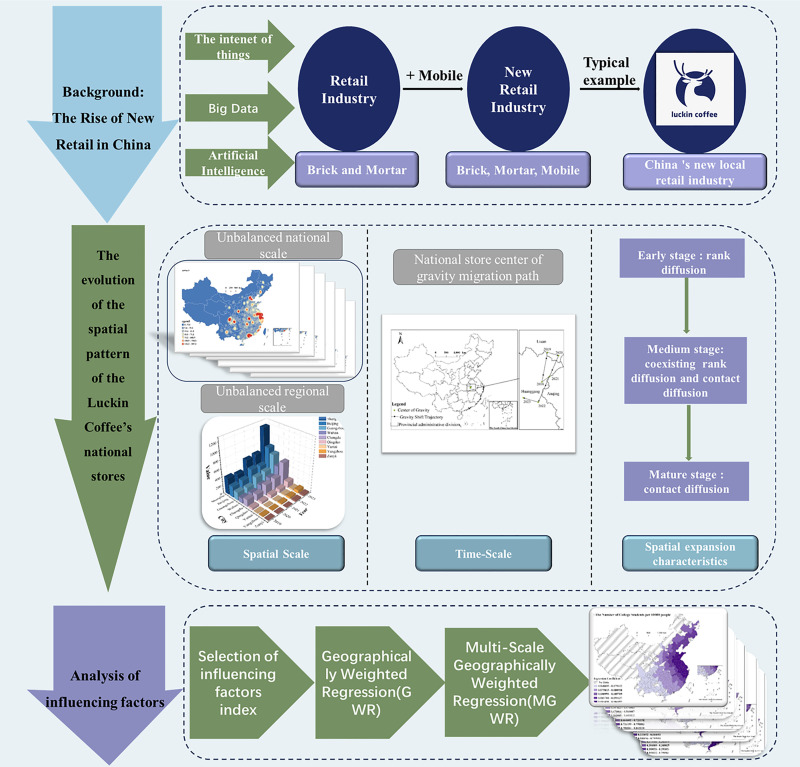
Framework of the spatio-temporal expansion patterns and influencing factors of Luckin Coffee in China. (Note: The figure is drawn based on the standard map from the Standard Map Service Website of the Map Technical Review Center of the Ministry of Natural Resources (Map Review Number: GS(2023)2767), and no modifications have been made to the base map boundaries.).

Scholars have interpreted the connotation characteristics of new retail and proposed that “omni-channel, digital operation, intelligent store, socialized goods, and efficient logistics” are the five major characteristics of new retail, that is, “data-driven, channel integration”, creating a new format in the retail consumption field [34]. Generally speaking, new retail is the integration of online and offline combined with high-efficiency logistics distribution, which is a close combination of the three, emphasizing consumers’ shopping experience, and redefining the retail format, consumer groups, technology, and brands around the three forms of “people, goods, and venue”. The research object of this article-Luckin Coffee, through in - depth analysis and mining of massive amounts of user purchase data, accurately discerns consumers’ preferences and behavior patterns. Consequently, it achieves fine - grained management in operational aspects such as product research and development, store location selection, and marketing promotion, earnestly implementing the concept of “digital operation” in the new retail industry. In the upsurge of the vigorous development of the new retail industry, numerous brands leverage advanced technologies such as the Internet and logistics to continuously explore and achieve self - innovation in the new retail format. Among them, Hema Fresh under Alibaba Group, JingDong’s 7FRESH, and Suning.com are all typical representatives in this field. Luckin Coffee, as an emerging force in the tea - beverage industry, has demonstrated a unique development trajectory in the practice of the new retail model. Luckin Coffee focuses on the segmented tea - beverage sector. With its relatively low production - scale costs, it has shown a strong momentum of explosive growth. By the first quarter of 2024, Luckin Coffee had opened more than 18,000 stores nationwide, with a rapid expansion trend. This remarkable development achievement has enabled Luckin Coffee to reach a relatively high level of penetration in the national market, providing sufficient sample data for this study. As shown in Fig 1, this study analyzes the temporal and spatial expansion patterns and spatial structure mechanisms of the new domestic retail brand, Luckin Coffee, from a geographical perspective on a national macro scale. It seeks to empirically supplement existing spatial expansion patterns of new retail brands in China and provide practical validation for theories regarding corporate spatial expansion. This research is crucial for understanding the spatial expansion dynamics of this new retail industry and enriching case studies in business and urban geography.

## Materials and methods

### Study area

The study area included 31 provinces (autonomous regions and municipalities directly under the central government) in China, excluding Hong Kong, the Macao Special Administrative Regions, and Taiwan Province. For the impact factor analysis, prefecture-level and above cities were used as the basic research units, due to data availability.

### Data sources

This study primarily focuses on 367 prefecture-level and above administrative regions in China as the research area and examines Luckin Coffee stores as the research subject. The point of interest (POI) data were sourced from Baidu map for the years 2018–2023. After processing for duplicate value elimination, anomaly screening, and coordinate correcting, the final extracted valid information amounted to 4042, 4351, 3759, 5752, 8729, and 15416 POIs, respectively. To further test the reliability of the data, first, the POI data of each year were imported into ArcGIS 10.8. Its spatial distribution in each city space was observed, revealing no obvious spatial discontinuity. Additionally, the number of POIs for Luckin Coffee stores in the study was compared with the number officially released by Luckin, except that the official statistical data was not released in 2018, the number of Luckin Coffee stores in 2019, 2020, 2021, 2022, and 2023 is 4507, 3929, 6024, 8214, and 16248 respectively, which shows a relatively small difference. This suggests that the data’s reliability is adequately reflected. Data on the impact factor indicators were primarily obtained from provincial and municipal statistical yearbooks, the statistical bulletin of the national economic and social development of cities, and the official local government websites for years 2021, 2022, and 2023. Considering the availability and completeness of data as well as the influence of cities, 341 prefecture-level cities were selected as research objects (excluding Hong Kong, Macao, Taiwan, and Sansha City), and areas with a POI value of 0 were excluded.

### Research methodology

#### GIS spatial analysis.

(1) Kernel Density Estimation (KDE) can be used to represent the spatial distribution density of Luckin Coffee stores. This method characterizes the spatial distribution of elements through morphological features, providing an intuitive reflection of how discrete measurements are distributed in a continuous area. KDE also helps to more clearly expresses the dispersed or discrete characteristics of the elements [35]. The method is implemented using the Kernel Density tool in ArcGIS 10.8 software.
(2) Centroid Migration is a statistical method used to analyze the spatial phenomena over time [36]. The migration distance and direction of the center of gravity were calculated by using the Mean Center tool in ArcGIS 10.8 software to compare the positions of the center of gravity at different time points. It reveals the spatial movement trajectory and changing trends of a phenomenon by calculating the position of the center of gravity at different time points.

#### Geo-detectors.

The factor detector in the Geodetector was used to assess the consistency of the spatial distribution patterns between the dependent and independent variables. Based on this consistency, the degree of explanation of the independent variable to the dependent variable- that is, the size of the q-value and the level of significance- was measured using the following formula [37]:


$$q=1-\frac{\sum_{h=1}^{L}N_{h}\sigma_{h}^{2}}{N\sigma^{2}}$$

where the value range of *q* is [0, 1]; the larger the value of *q*, the stronger the explanatory power of the independent variable *X* on the spatial differentiation of Luckin coffee, and vice versa. Where ℎ is the category index of a factor, *L* is the number of categories in which a factor is categorized, $N_{h}$ is the number of samples in the ℎ^th^ category, and N is the total number of samples. $\sigma_{h}^{2}$ is the variance of the target variable in the ℎth category, and $\sigma^{2}$ is the variance of the overall sample.

#### Multi-scale geographically weighted regression (MGWR).

The MGWR is used to determine the spatial heterogeneity of the regression coefficients across different independent variables [38]. This method allows for a more precise representation of the influence that each factor exerts the distribution density of Luckin Coffee across different districts. As an improvement of the classical geographically weighted regression (GWR), the largest difference between the MGWR lies in the heterogeneity of the bandwidth. In MGWR, the golden search algorithm can be used to identify the most suitable bandwidths for different variables, thus enabling more accurate regression modeling by avoiding the mismatch between the bandwidths and scales of variable effects. The model was formulated as follows:


$$\begin{array}{c} y_{i}\;\;\;\;\;\;\;\;\;=\beta_{0}(u_{i},v_{i})+\sum_{k=1}^{p}\;\beta_{k}(u_{i},v_{i})x_{ik}+\in_{i} \end{array}$$

where $y_{i}$ is the dependent variable for location i, $x_{ik}$ is the *k*^*th*^ explanatory variable for location i, $\left(u_{i},v_{i}\right)$ are the coordinates of location i, $\beta_{k}(u_{i},v_{i})$ are the regression coefficients of the *k*^*th*^ explanatory variable for location i, which are spatially varying, and $\in_{i}$ is the error term.

## Evolution of the spatio-temporal distribution pattern of Luckin Coffee in China

### Spatial distribution of Luckin Coffee in China

**Fig 2:**
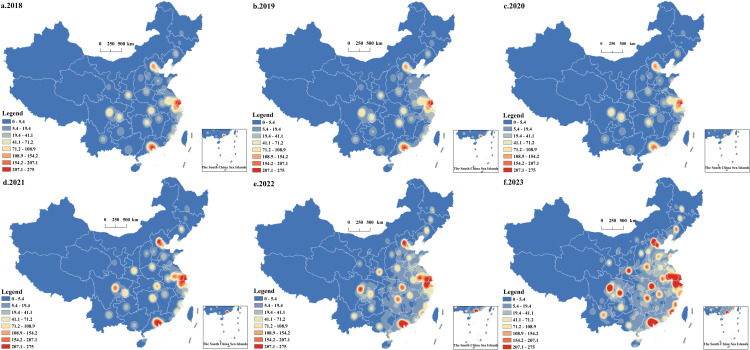
Spatial differentiation of kernel density index of Luckin coffee from 2018 to 2023. (Note: The figure is drawn based on the standard map from the Standard Map Service Website of the Map Technical Review Center of the Ministry of Natural Resources (Map Review Number: GS(2023)2767), and no modifications have been made to the base map boundaries.).

As shown in Fig 2, the spatial concentration area of Luckin Coffee stores in China reveals a spatial pattern and evolution trend of gradual expansion from the eastern coast to the western inland. These stores are predominantly located in the economically developed areas along the eastern coast, provincial capitals, municipalities, and urban agglomerations. Since 2018, the imbalance in the distribution of Luckin stores has eased, with the difference between the north and south, as well as the east and west, gradually decreasing. The overall direction of distribution now follows “northeast-southwest” axis. China’s urban agglomerations are the primary areas for the spatial agglomeration of Luckin Coffee stores and play a leading role in the its evolution. These agglomerations remain at the forefront of spatial concentration. During the study period, the distribution of high-value areas of spatial agglomeration of Luckin Coffee stores was relatively stable, with a continuous distribution from north to south, mainly in the urban agglomeration areas of Beijing-Tianjin-Hebei, Yangtze River Delta, Chengdu-Chongqing urban agglomeration, West Coast of the Taiwan Straits, and the Guangdong-Hong Kong-Macao Greater Bay Area, with urban agglomerations of the coastal areas playing a leading role, especially in the urban agglomerations of Beijing-Tianjin-Hebei and the Yangtze River Delta, which showed stronger spatial contiguous development trends. It should be noted that, as national-level city clusters, Liaozhongnan City Cluster, Central Plains City Cluster, and Shandong Peninsula City Cluster do not have outstanding performance in the process of Luckin Coffee store agglomeration. Although the spatial contiguous development trend of the Luckin Coffee store resources of the above city clusters is weaker, it still shows the patterns of clustering with the provincial capital cities as the core, gradually extending neighboring cities. This pattern reflects a “single-core” spatial structure.

**Fig 3:**
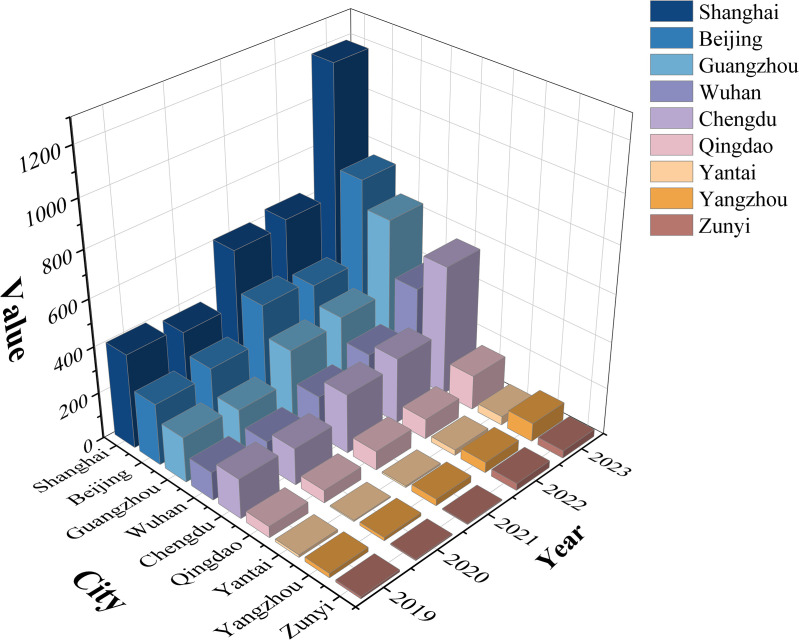
Changes in the number of luckin coffee in typical cities of the first, second and third tiers.

According to Fig 3, the change in the number of Luckin Coffee stores across the above nine cities between 2019 and 2023, categorized into first-tier cities (Shanghai, Beijing, and Guangzhou), second-tier cities (Chengdu, Wuhan, and Qingdao), and third-tier cities (Yangzhou, Yantai, and Zunyi), demonstrates a significant difference in expansion across different tiers of cities. From a comprehensive perspective, the number of Luckin Coffee stores shows a decreasing pattern from the first-to third-tier cities, which is closely related to the economic, market, and consumption capacities of each city. As economic and cultural centers, first-tier cities have higher consumption capacity and greater market demand, which attracts more Luckin Coffee stores. Although second-tier cities are not as large as first-tier cities, they have also become important markets for Luckin Coffee expansion owing to rapid economic development and rising consumption levels. In third-tier cities, economic and market constraints have caused the expansion of Luckin Coffee to be relatively slow. Additionally, from a temporal perspective, the number of Luckin Coffee stores in each city increased between 2019 and 2023, with particularly significant growth between 2022 and 2023. This may be due to the combined factors of Luckin Coffee’s brand influence, its market strategy, and changes in consumption habits. In conclusion, by analyzing the changes in the number of Luckin Coffee stores in first-tier, second-tier, and third-tier cities between 2019 and 2023, one can clearly observe the expansion path and strategy of Luckin Coffee in the Chinese market. This reflects the significant impact of the city’s economic level and market demand on Luckin Coffee’s expansion.

**Fig 4:**
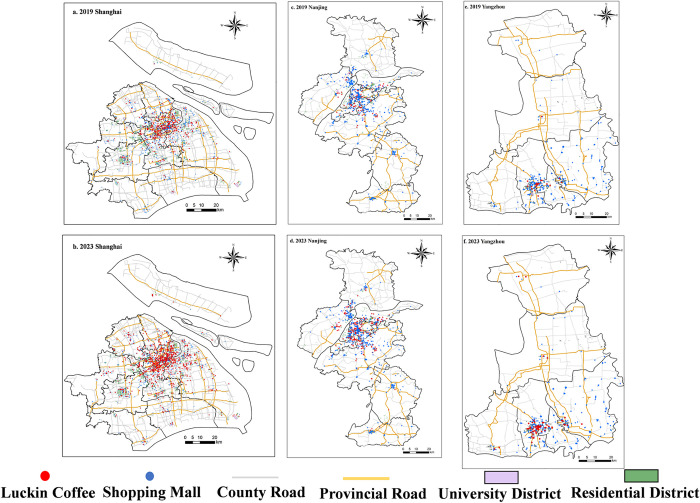
Spatial Distribution of Luckin Coffee within Cities. (Note: The figure is drawn based on the standard map from the Standard Map Service Website of the Map Technical Review Center of the Ministry of Natural Resources (Map Review Number: GS(2024)0650), and no modifications have been made to the base map boundaries.).

In order to study the spatial expansion characteristics of Luckin Coffee within different levels of cities, this paper selects two time nodes, 2019 and 2023, and three case sites, first-tier city-Shanghai, second-tier city-Nanjing, and third-tier city-Yangzhou, and establishes a spatial distribution database of Luckin Coffee in the urban areas of the three cities based on Point of Interest (POI) data. Shanghai, Nanjing, and Yangzhou are all situated within the Yangtze River Delta region, sharing similar geographic proximity and natural environmental conditions. By selecting these three cities as case studies, the potential influence of regional variations is effectively minimized, thereby providing a more robust basis for examining Luckin Coffee’s expansion strategies and market adaptability across different tiers of cities. As can be seen in Fig 4, from a common point of view, Luckin Coffee stores are centrally located in the main urban area, forming a cluster, and then spreading to the neighboring sub-districts and counties, in a stepwise and gradual layout, presenting the spatial form of “one nucleus and multiple levels”, and there is a big difference in the number of Luckin Coffee stores in various districts. Considering the density of consumer groups, the population is concentrated in the main urban areas, with a large flow of people and a stable source of customers. Moreover, transportation convenience is a key factor. There are numerous transportation hubs in the main urban areas, and at the same time, the distribution efficiency is higher in the central areas, which conforms to Luckin’s takeout business strategy. Short-time delivery can ensure the quality of beverages, enhance the consumption experience, and attract more customers who rely on takeout. In terms of differences, the development in first-tier cities is more rapid. The expansion of Luckin Coffee stores has gradually penetrated from the main commercial and supermarket areas into residential areas and the surrounding areas of university towns. The distribution of coffee shops in urban areas shows a developmental process from points to lines and then to surfaces. In contrast, in Nanjing (a second-tier city) and Yangzhou (a third-tier city), the development pace is slightly slower, and the spatial expansion trend is weaker. However, with economic development, the growth of young consumer groups, and Luckin Coffee’s continuous penetration through marketing, the number of stores is still on the rise. For Luckin Coffee, as a new retail industry, its development mainly relies on strong distribution support, high acceptance and purchasing power of consumers, and also requires a solid supply chain to maintain operations. Therefore, it cannot achieve large-scale layout in a short period of time. Deep cultivation in the same region is conducive to further expanding its brand influence, consolidating the market, completing the primitive accumulation, and quickly achieving profitability without expanding the supply chain.

### Temporal dynamic expansion of Luckin Coffee in China

**Fig 5:**
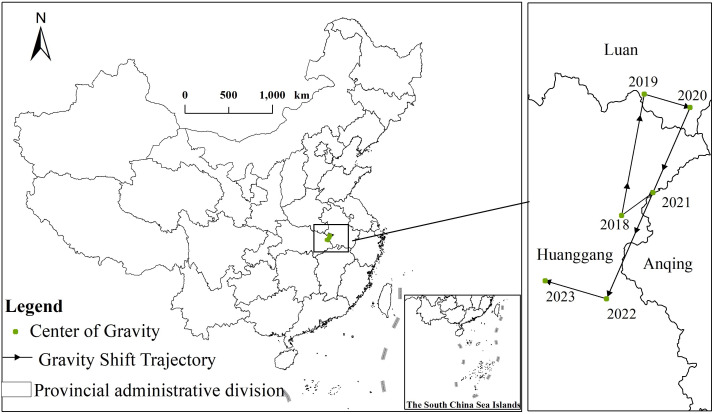
The center of gravity migration trajectory map of Luckin coffee from 2018 to 2023. (Note: The figure is drawn based on the standard map from the Standard Map Service Website of the Map Technical Review Center of the Ministry of Natural Resources (Map Review Number: GS(2023)2767), and no modifications have been made to the base map boundaries.).

According to Fig 5, the geographic coordinates of the center of gravity lie within the range of 115.50°–116.02°E and 30.60°–31.10°N, with the overall migration from northeast to southwest. During the study period, the center of gravity primarily moved between Huanggang City in Hubei Province and Lian City in Anhui Province. The distance of movement in the north-south direction was much greater than in the east-west direction. From 2018 to 2019, the center of gravity moved northward to Liuan City, Anhui Province. From 2019 to 2020, it shifted slightly southeast within Anhui Province. From 2020 to 2022, the center of gravity continued its southeastward movement Anhui Province. From 2020 to 2022, the center of gravity continued to migrate substantially to the southwest, eventually returning to Huanggang City in Hubei Province. In the same period, it then continued to migrate to the northwestern part of Hubei Province. Over the past five years, the expansion of Luckin Coffee stores has primarily focused on the southwest, gradually advancing from the central region to the southwest cities.

From 2018 to 2020, the center of gravity moved northward from Huanggang City to Liuan City. The main reasons for this movement can be analyzed from two aspects.Firstly, market potential: Large cities in the north and central regions (such as Beijing, Tianjin, Zhengzhou, etc.) have greater market potential and higher consumption levels, making them suitable for initial expansion of the key areas.Secondly, brand establishment: The centralized layout of stores in these large cities helps Luckin Coffee to quickly establish brand awareness and market influence, attracting more consumers and investors. Starting in 2020, the center of gravity gradually moved inland to the west. This stage of migration indicates that Luckin Coffee is gradually expanding its store coverage to the western region, further penetrating second- and third-tier cities. The primary reasons can be analyzed from the following four aspects. First, market saturation: After reaching a certain number of stores in major cities in the north and central China, the market may become saturated, necessitating new growth points. Second, emerging markets: Second-tier and third-tier cities in Southwest China (e.g., Chongqing, Chengdu, Guiyang, etc.) have experienced rapid economic development in recent years. As the consumption level of the residents has improved, there has been an increase in the demand for consumer goods, such as coffee, making these areas new expansion targets for Luckin Coffee’s expansion.Thirdy: competition avoidance: Expansion westward can help avoid fierce competition in the north and central regions, especially in cities with numerous existing coffee brands, thus offering more opportunities by entering new markets. Fourth, policy support: Local governments provide supportive policies to emerging enterprises and brands, including tax incentives and investment subsidies, which further promote Luckin Coffee’s expansion in these regions. Additionally, the advancement of China’s Western development policy has significantly improved the infrastructure and business environment in the Western region, attracting more corporate investment.

The market layout strategy of Luckin Coffee can be better understood by analyzing the migration trajectory of the store’s center of gravity. Initially, Luckin Coffee focused on rapid expansion to establish a broad coverage and create a brand effect. Once a solid foundation of the brand was established, the company gradually expanded toward decentralization, entering more emerging markets to achieve sustainable growth. The changes in expansion strategy not only reflect the changes in market demand and competitive environment but also show Luckin Coffee’s keen grasp of market opportunities and flexible response.

### Characterization of spatial expansion

The expansion mode of Luckin Coffee at different stages reflects the combined application of rank diffusion and contact diffusion. Its spatial expansion mode has undergone three stages: rank diffusion-based, coexisting rank diffusion and contact diffusion, and contact diffusion-based. In the initial and expansion stages, hierarchical diffusion was the primary strategy for establishing a brand base by entering first- and second-tier cities with high spending power. In the mature stage, the role of contact diffusion increases gradually, with the brand expanding rapidly in more second- and third-tier cities through consumer word-of-mouth and social media. This diffusion pattern is closely related to the general law of the geographic diffusion of corporate economic phenomena and the diffusion theory of cultural institutions, reflecting the successful expansion path and strategy of Luckin Coffee in the Chinese market.

In the early stage of its establishment, Luckin Coffee needed to rapidly increase its brand awareness and market penetration. Thus, it primarily focused on first-tier cities and some second-tier cities with more developed economies, such as Beijing, Shanghai, Guangzhou, and Chengdu. These cities, characterized by spatial expansion in the form of hierarchical diffusion, high spending power, dense population, and high brand acceptance, provide efficient marketing and consumer base, making them ideal for new brands to build their market presence. In the expansion stage, its spatial expansion exhibited a clear mix of hierarchical diffusion and contact diffusion. Meanwhile, Luckin deployed its spatial expansion in a leapfrog fashion according to the hierarchical scale of cities, entering first-tier provincial capitals (e.g., Beijing, Shanghai, Guangzhou) for the first time. However, after establishing the key provincial capitals, Luckin rapidly expanded to more second-tier cities (e.g., Wuhan and Qingdao) and some potential third-tier cities (e.g., Yangzhou and Yantai) through contact diffusion. The increase in brand awareness and expansion of the user base allowed Luckin coffee to enter new markets through word-of-mouth (contact diffusion) among users. Meanwhile, Luckin coffee has attracted more consumers in both second- and third-tier cities by continuously optimizing its products and services. In the mature stage, the expansion presents as a spatial expansion characterized by contact diffusion. The number of stores shows exponential growth, particularly with the significant increase in these second- and third-tier cities, where the company has developed a stable customer base. This growth is attributed to the brand’s established high level of awareness and reputation, which, coupled with, word-of-mouth promotion among existing consumers and the extensive influence of social media, has accelerated the company’s expansion into new markets. At this stage, Rexall established an efficient logistics and supply chain system nationwide, ensuring that new stores could quickly open and maintain a stable supply of products. This efficient operational model supported its rapid expansion across cities in all tiers. Additionally, the support of digitalization and online operations has allowed Luckin Coffee to interact directly with consumers and drive sales through online platforms (such as apps). This approach not only improves operational efficiency but also enhances the effect of contact diffusion through digital means. Consumers promote brand expansion by sharing their consumption experiences and discounting information.

## Analysis of the influencing factors in the spatial distribution of Luckin Coffee

### Indicator selection of impact factors

Geographical nature refers to the unique and essential characteristics of a region within geographical space [39]. In 1993, Krugman [40] proposed the theory of geographical nature to explain the phenomena of economic agglomeration and regional development. He suggested that there are two geographical natures that guide the evolution of geographical structure: natural endowment, referred to as the first nature, and human choice, aggregation, and location, referred to as the second nature. In the 21st century, new factors such as informatization, science, and technology have become increasingly significant for regional development. Liu Qingchun [41] identified human capital as the third type of geographic factor in the new economic environment, while Wang Zheng [42] proposed the condition of informatization as the third geographical factor and emphasized that developing the second and third geographical factors is key to overcoming the limitations of the first geographical factor and achieving geographic agglomeration. Currently, academics have conducted numerous studies on the influence of geographic nature on the mechanism of business resource agglomeration [43,44]. Luckin Coffee, as an emerging resource element developed in the big data era, introduces geographic nature as the theoretical basis for constructing an indicator system with certain exploratory value and theoretical significance.

This study explored the factors influencing the spatial distribution of Luckin coffee stores in China from the perspective of the geographical nature of the region. Following the principles of indicator scientificity and data availability, and drawing on existing studies [43,45], average temperature, air quality, and environmental protection capacity were selected as the main indicators characterizing the first geographic nature; consumption capacity, spatial capital, urban construction level, and market size were selected as the main indicators characterizing the second geographic nature; and informatization level, innovation capacity, human capital, and cultural resources were selected as the main indicators characterizing the third geographic nature (Table 1).

**Table 1:** Influencing factors and indicators.

Geographical Nature	Variable	Influencing Factor	Selection of Specific Indicators
The first geographical nature	X1	Average temperature	Average annual temperature
	X2	Air quality	Air quality index (AQI) reached and exceeded the second-level days
	X3	Environmental protection capacity	Greening coverage in built-up areas
The second geographical nature	X4	Consuming capacity	Disposable income of all residents
	X5	Spatial capital	Bus ownership per 10,000 people
	X6	Urban construction level	Urbanization rate
	X7	Market size	Natural growth rate of population
The third geographical nature	X8	Informatization level	Internet penetration rate
	X9	Innovation ability	Patent ownership per 10,000 people
	X10	Human capital	Number of college students per 10,000 people
	X11	Cultural resource	Public library collections per 10,000 people

### Geodetector-based analysis of influencing factors

Statistical data for 2021, 2022, and 2023 were selected to analyze the factors influencing the evolution of the spatial pattern of Luckin Coffee using geodetectors. The selected indicator X (independent variable) was stratified using Natural Breaks to convert it from a numerical to a typological quantity.

As presented in Table 2, from 2021 to 2023, except for the average temperature (X1) and air quality (X2), the q-value change of each influencing factor generally demonstrates a gradual increase, following the “low-high” trend, with the highest value observed in 2023. This indicates that the spatial distribution of Luckin Coffee stores is increasingly influenced by various factors. In terms of the ranking of the q-value mean, the top four indicators among the 11 analyzed are urban construction level (0.464), consumption capacity (0.355), informatization level (0.338), and human capital (0.225). This indicates that the spatial distribution of Luckin Coffee stores is greatly influenced by the second and third geographical nature. During the study period, the first factor (geographical nature) remained consistently in the lowest three places in the ranking of influencing factors. Overall, these three factors had a relatively small impact on the spatial distribution of Luckin Coffee.

**Table 2:** Geographical detection results of influencing factors of spatial distribution of Luckin coffee.

Q-Value	X1	X2	X3	X4	X5	X6	X7	X8	X9	X10	X11
2021	0.042	0.132	0.076	0.187	0.113	0.335	0.086	0.196	0.123	0.163	0.123
2022	0.11	0.123	0.097	0.385	0.228	0.405	0.122	0.367	0.157	0.211	0.178
2023	0.056	0.129	0.135	0.493	0.287	0.651	0.225	0.452	0.299	0.301	0.192
Mean Value	0.069	0.128	0.103	**0.355**	0.209	**0.464**	0.144	**0.338**	0.193	**0.225**	0.164
Ranking	11	9	10	2	5	1	8	3	6	4	7

Further analysis of the q-values of the above significant factors reveals that the second geographical nature is the dominant factor in the spatial distribution of Luckin Coffee stores. The explanatory power of the city construction level and consumption capacity ranks first and second, respectively, significantly higher than other influencing factors. The is because the urban construction level reflects the comprehensive strength of the city, which is the basis for ensuring the construction of commercial infrastructure and the effective supply of resources. Consumption level of the residents plays an economic support role, affecting the level and capacity of regional consumption and generating a stronger attraction for the agglomeration of Luckin Coffee stores. In addition, the level of informatization and human capital in the third geographical region also play varying roles in promoting the clustering of Luckin Coffee resources.

### MGWR-based spatial differentiation of influencing factors

**Fig 6:**
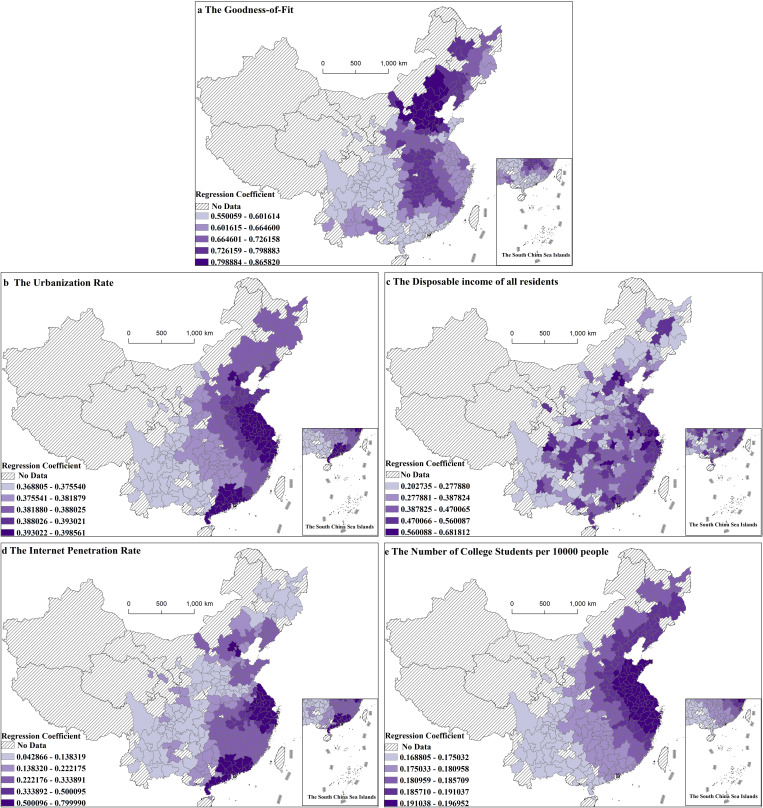
Spatial distribution of regression coefficients of influencing factors in MGWR mode. (Note: The figure is drawn based on the standard map from the Standard Map Service Website of the Map Technical Review Center of the Ministry of Natural Resources (Map Review Number: GS(2023)2767), and no modifications have been made to the base map boundaries.).

To further explore the spatial difference characteristics of the influencing factors of Luckin coffee store distribution, this study employed the MGWR model to analyze the data for 2022. The POI value of Luckin Coffee stores was used as the dependent variable, and the influencing factors with the top four explanatory power rankings in each dimension were selected as the independent variables. Geographically weighted regression analyses were conducted from a local perspective. The results, which passed the hypothesis test with a confidence level of 95% for each factor, were visualized and expressed through the natural breakpoint grading method (Fig 6). Among the model parameters, the number of effective parameters was 261 (removing the areas with zero POI value), the Akaike information criterion AICc was 593.489, and the goodness of fit R2 was 0.712, indicating a good fit and reflecting credible geo-detector results. Table 3 shows that the regression coefficients of each influencing factor were significant, indicating clear spatial differences in the effects of all four independent variables on the spatial distribution of Luckin coffee.

**Table 3:** Statistical description of regression coefficients of MGWR model.

Variable	Bandwidth	Mean Value	Standard Deviation	Minimum Value	Median	Maximum Value
Urbanization rate	351	0.385	0.008	0.369	0.386	0.399
Disposable income of all residents	137	0.373	0.132	0.203	0.387	0.682
Internet penetration rate	351	0.265	0.194	0.043	0.213	0.800
Number of college students per 10,000 people	70	0.183	0.008	0.169	0.184	0.197

In the results of the MGWR regression analysis (Table 3), the regression coefficients of each influencing factor in each unit of analysis were counted to obtain their mean, standard deviation, minimum, median, and maximum values, which showed that each influencing factor differentiated the spatial distribution of Luckin Coffee stores.

The local R2 for the sample analysis units were all greater than 0.55 (Fig 6a), indicating that the overall goodness of fit is better. There is a significant north-south difference in R2, with Beijing-Tianjin-Hebei region, northeastern region, and eastern coastal region exhibiting larger values of goodness-of-fit. This indicates that the constructed MGWR model has a greater explanatory power for the above regions, while the Hainan region, most of the regions of Guangdong and Guangxi, and the inland regions of the central and western parts of the country have relatively lower values of goodness-of-fit. Conversely, Hainan, Guangdong, most of Guangxi, and inland areas in the central and western regions have relatively low goodness-of-fit values, indicating that these regions are affected by other factors in addition to the variables of the constructed model.

The urbanization rate has a significant positive effect, with the difference between the maximum and minimum regression coefficients of only 0.02976. This indicates that Luckin Coffee stores are primarily distributed in areas with high urbanization. As illustrated in Fig 6b, the regression coefficient of the urbanization rate showed a trapezoidal decrease in distribution from the east coast to the west inland. High level of urbanization create an business ecosystem and resource allocation for Luckin Coffee. The region usually has a more complete business ecosystem with large shopping malls, office buildings, and residential communities, which provides Luckin Coffee with diversified site selection opportunities. Coffee’s flexible business model (e.g., small stores and pick-up points) can be adapted to different business environments and quickly capture the market. Urbanization also promotes the rational allocation of resources in a region, including human, capital, and technical. The effective allocation of these resources helps improve the operational efficiency and service quality of Luckin Coffee.

The disposable income of all residents significantly and positively affects the spatial distribution of Luckin coffee stores; the regression coefficient value is generally larger than that of other influencing factors, the difference between the maximum and minimum regression coefficients is 0.4790, and the spatial non-equilibrium is relatively obvious (Fig 6c). The disposable income of all residents greatly affects the residents’ consumption level, and a higher disposable income of consumers indicates that they have stronger economic payment ability and willingness to pay; therefore, the disposable income of the whole population plays an economically supportive role in the evolution of the agglomeration of commercial resources, such as Luckin Coffee. Regional consumption ability and level also affect the direction of the flow of resource elements, and regions with a high level of consumption are more capable of meeting the convergent demand for commercial resources. Regional consumption capacity and level also affect the flow direction of resource factors, and regions with high consumption levels are more capable of meeting the demand for commercial resources, which in turn creates stronger attraction for the agglomeration of commercial resources.

The difference between the maximum and minimum values of the regression coefficient of internet penetration was 0.7571, indicating large differences in internet penetration across regions. The highest regression coefficients were found in the Beijing-Tianjin-Hebei, Yangtze River Delta, and Guangdong-Hong Kong-Macao Greater Bay Area urban agglomerations (Fig 6d), indicating that the Internet penetration rates in these regions are highly consistent with the number of Luckin Coffee stores. Regions with high internet penetration have a high acceptance of online ordering, mobile payment, and other modes, which can better support Luckin Coffee’s online and offline integration operation modes and improve operational efficiency and customer satisfaction. Additionally, Luckin Coffee can improve distribution efficiency and service quality by optimizing its distribution network, such as real-time tracking and intelligent scheduling. It can also accurately grasp consumer demand through Internet data analysis for personalized marketing and product optimization.

The number of university students among 10,000 people is positively correlated with the distribution of Luckin coffee stores (Fig 6e), and the difference between the maximum and minimum regression coefficients is only 0.0281, indicating that Luckin coffee stores across China are mostly located in areas with a sufficient number of university and college students. This provides a broad consumer market for them. Human capital and degree of openness played a significant role in the agglomeration evolution of Luckin coffee stores. The high consumption concepts, economic capital, and willingness of highly educated talent to accept new things have promoted the diversification of commercial resources in the region. The primary consumer group in the tea and coffee market is young people, who have diverse needs for beverages and are willing to try new flavors and brands, driving the rapid growth of the market and making university campuses and the surrounding business districts important markets for Luckin Coffee. Through optimizing store location, product, and service strategies, Luckin Coffee can better meet the needs of young consumer groups and drive the aggregation of store resources and business growth.

## Discussion

The new retail model transforms and enhances the traditional business operation mode through the Internet, achieving an optimal equilibrium between product price and convenience by seamlessly integrating “self-pickup+takeout” and “offline+online.” Therefore, this business format differs significantly from traditional retailing, and the influence of spatial constraints has progressively diminished. The layout characteristics of the new retail industry are, to a certain extent, a revision of the technological revolution within the center-ground theory in retail analysis. From a macroscopic spatial scale perspective, new retail exhibits significant central - orientation, mainly concentrated in economically developed coastal cities and first - tier core cities. However, there are still substantial differences in the specific spatial expansion patterns and location - selection mechanisms between new retail and traditional retail. On the one hand, the expansion process of new retail is extremely rapid. The cycle of transformation from rank diffusion to contact diffusion is significantly shortened, enabling explosive growth in the number of stores over a large area within a relatively short time span, and the spatial distribution is more uniform and homogeneous. On the other hand, when focusing on the expansion patterns within different - level cities, it can be clearly observed that the degree of dependence of new retail on urban central areas is significantly lower compared to traditional retail businesses. In this development process, the important position of geographical centrality is gradually replaced by logistics centrality to a certain extent. The spatial constraints faced by the retail industry are continuously weakening, thus presenting the typical characteristic of “infinite scenarios”. This phenomenon of obvious differences from the location - selection strategies of traditional retail is not solely due to the impact of information technology innovation on the location - selection strategies of the retail industry. More crucially, the profound transformation of business concepts and service concepts in the new retail era has jointly promoted the systematic transformation of retail location - selection strategies.

With the in-depth development of advanced technologies such as big data and artificial intelligence, the types of retail industry characterized by new retail models have continued to grow, and numerous retail stores following these models have emerged, providing valuable research opportunities for studying the differences in location choice and spatial association characteristics between new retail stores of different types, brands, and traditional retail stores. Some studies compare Starbucks as a representative of traditional retail, with Luckin Coffee, a representative of new retail, to analyze the spatial distribution characteristics and site selection criteria of new retail [2]. Luckin Coffee adopts a “dual-line” consumption model to provide convenient services, which refers to the sales model of “online + offline + delivery.” This promotes a high degree of integration between online and offline, optimizes both its e-commerce platform and offline physical stores, meets consumer needs in various scenarios, and realizes the “infinite scenarios” of coffee consumption. In contrast, Starbucks follows the business philosophy of the “third space,” where its products combine coffee and coffee culture, the third space culture. Its consumption scene is “coffee + office + leisure + study.” The “infinite scene” and the “third space” represent the two business formats of new retail and traditional retail. Studies have been conducted to explore the spatial diffusion characteristics and influencing factors of Starbucks in mainland China at a national macro scale [46]. However, empirical studies on specific brands of the new retail industry at the macro scale are rare, which is not conducive to exploring the spatial expansion dynamics and influencing factors of the new retail industry. Therefore, this study, drawing upon retail location theories and corporate spatial growth, uses the Luckin Coffee brand as a case study. Through obtaining multi-temporal cross-sectional data, this research quantifies the spatial expansion process of Luckin’s chain of stores, providing a detailed and quantitative analysis of the expansion process aimed at revealing the spatial growth and expansion mode of the Luckin Coffee-a key representative of the new local Chinese retail industry-on the macro level. Based on this study, future research could also consider the competitive influence of Starbucks, a similar traditional retail competitor in the same study area, and explore which model better aligns with the preferences of China’s new middle-class coffee consumers: “the infinite scenarios or the third space”? Additionally, as a highly influential retail brand, the expansion process of Luckin Coffee in the temporal dimension and its expansion pattern in the spatial domain are the result of the interplay of multiple factors. Among these, the franchising model implemented by the company, through the construction of a unique franchise system, plays a crucial role in aspects such as the speed of store expansion and the breadth of regional coverage. Numerous factors involved in the franchising and marketing models, such as the local resource integration capabilities of franchisees, the impact of marketing strategies on consumer psychology and behavior, and the dissemination effect of the brand image in different cultural and regional contexts, possess strong subjectivity and situational dependence. Thus, it is difficult to accurately measure them solely using quantitative analysis methods. Therefore, for these qualitative aspects that are difficult to quantify, it is urgent to conduct more in - depth and systematic discussions to comprehensively analyze the complex mechanisms underlying the spatio - temporal expansion model of the Luckin Coffee brand.

## Conclusions

This study analyzes the evolutionary characteristics of the spatial pattern of Luckin Coffee stores across the country from 2018 to 2023 using GIS spatial analysis and other methods. It also explores the factors influencing the spatial distribution of Luckin Coffee stores and their spatial differentiation characteristics using geo-detectors and MGWR models. The main conclusions of this study are as follows:

(1) Between 2018–2023, the agglomeration distribution pattern of national Luckin Coffee stores became increasingly pronounced, exhibiting expansion characteristics of extending from coastal regions to inland areas, predominantly in economically developed areas such as various urban agglomerations and first and second-tier cities. Through an analysis of Luckin Coffee’s center-of-gravity migration path, it was found that the company initially focused on rapid expansion to establish coverage to form a brand effect. After solidifying its brand foundation, it gradually expanded to decentralization. Luckin Coffee’s spatial expansion mode underwent three expansion stages: first, a hierarchical diffusion-based, followed by a co-existing hierarchical diffusion and contact diffusion phase, and finally, a contact diffusion-based phase, which follows the general law of enterprise expansion.
(2) According to the results of geographical detection, the explanatory power of each influencing factor on the spatial distribution of Luckin coffee shops varies significantly. The q-value changes of the influencing factors generally exhibit a gradual increasing trend of “low-high.” Here, the level of urban construction and consumption capacity categorized under second geographical nature, and the level of informationization and human capital, categorized under the third geographical nature, are the main influencing factors on the spatial distribution of Luckin Coffee.
(3) The MGWR model reveals that Luckin Coffee is concentrated in areas with high urbanization levels, and its influence decreases from east to west. The disposable income of residents significantly and positively affects the spatial distribution of Luckin Coffee stores, and the spatial imbalance is relatively obvious. Luckin Coffee is more reliant on Internet penetration in urban agglomerations such as Beijing-Tianjin-Hebei, the Yangtze River Delta, and Guangdong-Hong Kong-Macao Bay Area than in other areas. The number of university students per million is positively correlated with the distribution of Luckin Coffee stores. The number of college students per 10,000 people is positively related to the distribution of Luckin Coffee.

## Supporting information

S1 File Factors. (XLSX)

S2 File POI data. (XLSX)
